# Thermal effects on feeding efficiency and body condition in invasive and native benthivorous freshwater fishes

**DOI:** 10.1007/s10530-026-03767-w

**Published:** 2026-02-12

**Authors:** Christophe Benjamin, Jaclyn Hill, Anthony Ricciardi

**Affiliations:** 1 Department of Biology, McGill University, Montreal, QC Canada; 2 Bieler School of Environment, McGill University, Montreal, QC Canada; 3 Maurice Lamontagne Institute, Fisheries and Oceans Canada, Mont-Joli, QC Canada

**Keywords:** Biological invasions, Functional response, *Tinca tinca*, Impact prediction, Freshwater fish, Body condition

## Abstract

Predictive information on invasive species impacts under climate warming is critical for risk assessment. The comparative functional response approach—an experimental method measuring feeding efficiency of a predator along a prey gradient—can forecast ecological impacts of an invader by quantifying its resource consumption under varying conditions. This approach was applied to the Tench *Tinca tinca*, an invasive benthivorous fish currently spreading in the St Lawrence River, and two native benthivorous species, the White Sucker *Catostomus commersonii* and the Brown Bullhead *Ameiurus nebulosus*. Body condition was used as a measure of long-term fitness under a given temperature treatment, and the Functional Response Ratio (FRR) and Relative Impact Potential (RIP) were used as metrics describing trophic impact. All species were acclimated to 18 °C and 25 °C; the former temperature represents the physiological optimum for the chosen native species, whereas the latter temperature is the projected near-future mean summer maximum for the lower Great Lakes. Feeding rates on larval chironomids were measured in 3-h trials at prey densities ranging from 2 to 500. White Sucker feeding efficiency was unaffected by temperature and consistently higher than the Tench, whereas the Tench exhibited a lower maximum feeding rate at 25 °C compared to 18 °C. Despite superior feeding rates, White Suckers showed diminished body condition at the elevated temperature, suggesting their foraging advantage might not persist under warming conditions. These findings suggest Tench possess greater thermal compensation capacity than White Suckers, indicated by their stable condition factors at elevated temperatures despite lower maximum feeding rates. In a second set of experiments, Tench achieved greater feeding efficiency than Brown Bullhead, but both species showed reduced efficiency at elevated temperatures. However, Brown Bullhead had increased body condition indices following experiments. FRR and RIP metrics indicated that Tench holds a competitive feeding advantage over Brown Bullhead that is reduced under elevated temperatures. Tench impact depends on both feeding efficiency and its capacity to maintain fitness in warm waters. Our results highlight the necessity of considering thermal adaptation in ecological forecasting. Climate warming may reshape competitive dynamics and ecosystem impact pathways beyond direct consumption effects.

**Supplementary Information:**

The online version contains supplementary material available at 10.1007/s10530-026-03767-w.

## Introduction

The colonization success and impact of an invading species are highly context dependent and can vary substantively across space and time (Catford et al. 2022). The Environmental Matching Hypothesis predicts that invading species will have greater per capita effects in novel environments in which a major limiting physicochemical variable, such as water temperature, is a closer match to their physiological optimum (Iacarella et al. 2015). Increased surface water temperatures due to climate warming will enable colonization of new habitats and enhanced performance in conditions previously suboptimal or unsuitable for warm-adapted species (Galil et al. 2007; Hellmann et al. 2008; Iacarella et al. 2015; Mofu et al. 2019). Access to resources will be mediated by new thermal regimes such that native species with narrow thermal tolerances can be excluded from warmer shallow habitats, whereas non-natives with wider thermal tolerance can thrive in such habitats (Tepolt and Somero 2014; Boddy and McIntosh 2017; Caldwell et al. 2020). Competitive dynamics between native and non-native species may also be affected when per capita consumption changes under elevated temperatures (Rahel and Olden 2008; Carmona-Catot et al. 2013).

Experimental measurements of an invader’s functional response (i.e. its rate of prey consumption as a function of prey density) across different environmental contexts can predict its trophic impacts in the field (Dick et al. 2013, 2014, 2017a, b; Alexander et al. 2015; Cuthbert and Briski 2021, but see Vonesh et al. 2017). A higher maximum feeding rate could indicate the potential for higher pressure on prey populations and greater resource competition with resident species of the same trophic level (Alexander et al. 2014). The shape (curve type) of the functional response describes how a predator’s consumption rate vary with prey density, which has implications for prey population stability. Type I responses exhibit linear increase of feeding rate in relation with prey density until a maximum is reached. In Type II responses, the attack rate (parameter *a*) is constant, and handling time (parameter *h*) limits maximum feeding rate such that an asymptote is evident. Type III responses differ by having an attack rate that varies inversely with prey density, generating a sigmoidal curve that provides a low-density refuge where predators are relatively inefficient. This refuge effect allows the prey to recover from low numbers, stabilizing predator–prey dynamics (Dick et al. 2013; Médoc and Spataro 2015). Functional response experiments can thus provide predictive information on how temperature influences feeding efficiency of sympatric predators with overlapping diets and its potential impact on prey population (Daugaard et al. 2019; Khosa et al. 2020; Uiterwaal and DeLong 2020).

Beyond comparing functional response curves directly, derived metrics can quantify the relative trophic impact of invasive versus native consumers. The Functional Response Ratio (FRR), calculated as the attack rate divided by handling time (*a/h*), provides a single metric of per capita feeding efficiency that facilitates comparisons across species and environmental conditions (Cuthbert et al. 2019). Higher FRR values indicate more efficient resource exploitation, as consumers locate prey more rapidly (higher *a*) or process prey more quickly (lower *h*). However, per capita effects alone do not capture population-level impacts, as these also depend on consumer abundance in the field. The Relative Impact Potential (RIP) integrates both per capita feeding rates and abundance of native and invasive populations to estimate comparative ecological impacts (Dick et al. 2017b). Calculated as the ratio of maximum feeding rates weighted by relative abundances between invasive and native species, RIP values greater than one suggest that the invasive species exerts a disproportionate impact on shared prey resources. These metrics provide a quantitative framework for predicting how shifts in temperature—by altering both feeding efficiency and population dynamics—may reshape competitive interactions and the ecological consequences of biological invasions.

Functional response experiments measure per capita consumption, a good indicator of relative feeding efficiency (Dick et al. 2014), but they do not measure fitness-related traits. Body condition indices provide essential proxies for assessing fish energetic status and fitness in different environments (Stevenson and Woods 2006). Multiple complementary metrics can be used to evaluate condition. The relative condition factor (Kn) compares an individual’s observed weight to the expected weight for its length based on population-specific relationships (Le Cren 1951), reflecting overall lipid reserves and energy content (Herbinger and Friars 1991; Mozsár et al. 2015). The hepatosomatic index (HSI), calculated as liver mass relative to total body mass, indicates hepatic lipid storage—a critical energy reserve in fish (Wootton 1989; Chellappa et al. 1995). The percentage of dry liver mass (%DML) quantifies lipid density within hepatic tissue, providing a direct measure of energy reserves that complements HSI (Wuenschel et al. 2019). Higher values of these indices are associated with enhanced reproductive success (Marshall et al. 1999; Brosset et al. 2016), improved overwinter survival (Pangle et al. 2004), and increased disease resistance (Neff and Cargnelli 2004). Species that maintain superior body condition under environmental stress may exhibit competitive advantages in invaded ecosystems, particularly as thermal regimes shift with climate change. Understanding how condition responds to temperature variation in invasive versus native species can therefore illuminate mechanisms underlying invasion success and ecological impact. In this study, we conducted functional response experiments to compare feeding efficiencies of the Tench *Tinca tinca* (Linnaeus 1758), a globally invasive Eurasian fish (Avlijaš et al. 2018), with two trophically similar native benthivorous species in the Great Lakes basin, the White Sucker *Catostomus commersonii* (Lacepède 1803) and the Brown Bullhead *Ameiurus nebulosus* (Lesueur 1819). The invasion success of the Tench is presumably facilitated by its broad environmental tolerance (Avlijaš et al. 2018), including a wide thermal range of 0–38 °C, with a preferred temperature range of 20–27 °C (Peňáz et al. 1989; Perez-Regadera et al. 1995). Over the last three decades in eastern North America, the Tench has spread from its original point of introduction in the Richelieu River to the St. Lawrence River, where it has increased its population size exponentially and now occupies most of the freshwater section of the river (Masson et al. 2013; Avlijaš et al. 2018). Further dispersal into Lake Ontario is expected, as it contains abundant wetland habitat that is ideal for feeding and reproduction of the Tench, which is a phytophilic spawner (Ashworth and Johnson 2021). The habitat and diet of both Tench and White Sucker substantially overlap (Becker 1983; Cudmore and Mandrak 2011; Ashworth and Johnson 2021). Continued range expansion of the Tench, coupled with projected climate warming (Trumpickas et al. 2009; Zhang et al. 2020), could potentially accelerate the ongoing decline in White Sucker populations in Lake Ontario (OMNRF 2021), especially as projected future surface water temperatures are expected to fall outside of the White Sucker’s optimal range (14–23 °C; Cincotta and Stauffer 1984; Hasnain et al. 2010; Richter et al. 2024). The Brown Bullhead also shares a similar diet and habitat with the Tench (Klarberg and Benson 1975; Kline and Wood 1996), but unlike White Sucker, its thermal optimum is comparable to Tench (23–30 °C; Crawshaw 1975; Richards and Ibara 1978; Keast 1985). This shared thermal optimum may influence coexistence with Tench in overlapping ecological niches, especially as both species are able to cope with extreme conditions by burying in the sediments Brown Bullhead (Baughman 1947; Becker 1983), and have shown to be globally invasive (Avlijaš et al. 2018; Collier et al. 2018; Francis 2019; Rechulicz and Płaska 2021; Ulikowski et al. 2022).

Here, we used a comparative functional response experimental design to evaluate feeding efficiencies of Tench, White Sucker, and Brown Bullhead under two temperature treatments, to test the following predictions:

1. Following the Environmental Matching Hypothesis, the feeding efficiency of each species will be higher at the temperature closer to their respective thermal optimum. Therefore, the maximum feeding rate (MFR) of the White Sucker will be higher at 18 °C than at 25 °C, whereas Tench and Brown Bullhead will have the opposite pattern.
2. Compared to the Tench, the MFR of the White Sucker will be higher Tench at 18 °C, but lower Tench at 25 °C. Brown Bullhead will exhibit a similar feeding efficiency to Tench at both temperature treatments.
3. The condition factors of each species will be higher at conditions closer to their thermal optimum.

## Methods

### Data collection

#### Fish capture and maintenance

Tench specimens were obtained from Lac St-Pierre, a fluvial lake of the St. Lawrence River (46.203326°, − 72.832405°), where they were captured by a commercial fisher using fyke nets (18m leader, 1.8 × 0.8 m, 4.4 cm mesh size) during June 24-July 7, 2021, at water temperatures of 18.9–21.3 °C (Canadian Coast Guard 2025) for the first set of experiments, and October 5–19, 2022, at 13.7–16.3 °C (Canadian Coast Guard 2025) for the second set of experiments. White Suckers were collected using a scientific weir net (83.1 m leader, 6.1 × 8.3 × 3.5 m, 3.1 cm mesh size) installed in the St. Lawrence River near Quebec City (46.741384°, − 71.294757°) and operated by the Québec Aquarium from August 5 to September 19, 2021, at 19.2–25.7 °C (Canadian Coast Guard 2025). Brown Bullheads were collected using the same fyke nets as Tench in Lac-St-Pierre and Richelieu River (45.137516°, − 73.256450°) from October 5 to November 21, 2022, at 2.7–16.1 °C (USGS 2025).

Captured individuals were collectively transported in 1000-L insulated totes (maximum 30 fish per tote) to the Maurice-Lamontagne Institute (Fisheries and Oceans Canada), where they were housed in 800-L tanks filled with dechlorinated water.During transport, water in the transport tanks was supplemented with sodium chloride (3 g/L), tricaine methanesulfonate (MS-222; 10 mg/L), Melafix^©^ (API Fishcare), and Vidalife^©^ water conditioner (Syndel). (Harmon 2009). Upon arrival, fish were treated for ectoparasites with two 1-h formalin baths two days apart. Holding and experimental tanks were each linked in two partially open-water systems (one for each temperature treatment) of 4000 L and 2000 L, respectively, with a water renewal rate of 10 L min^− 1^. Water temperature was regulated by a heat pump connected to the tank line, with a control accuracy of 0.5 °C. Temperature was recorded for each tank every 15 min using temperature loggers (2021: 18.10 ± 0.03 °C; 25.00 ± 0.04 °C; 2023: 18.10 ± 0.10 °C; 25.00 ± 0.19 °C; Onset HOBO Pendant MX2201). The photoperiod was set on 15:9 (photophase:scotophase) from 05:00 to 20:00, replicating local summer conditions. In 2023, tanks containing Brown Bullhead were covered in opaque material and lights were on from 1600 to 700 h, such that feeding and experiments were done during dark hours, the period when Brown Bullhead are most active (Eriksson and Veen 1980). Although Tench are also considered nocturnal (Siegmund 1969), no differences have been documented in food intake or growth performance between photophase and scotophase (Herrero et al. 2005), and White Suckers actively feed both day and night (Kavaliers 1980). Consequently feeding and experiments for Tench and White Sucker were completed during light hours. All tanks consisted of 4–8 fish, to reduce the stress of captivity. Adequate oxygen concentration was ensured by aeration of each tank and of the recirculated water in the head tank. Fish were acclimated at least for 28 days in the captivity tanks before the start of functional response trials. Two temperature treatments of 18 °C and 25 °C were chosen so that 1) White Sucker and Tench were subjected to experimental conditions within their respective optimal thermal ranges, and 2) the temperature contrast was large enough to expect an effect on feeding efficiency (Avlijaš et al. 2022; Hutchings et al. 2025; Claus et al. 2025). Fish intended for the 25 °C-treatment were first acclimated for 7 days at 18 °C, and then the temperature was raised by 1 °C·day^−1^ for 7 days, after which the fish were acclimated for 14 days at 25 °C. Salinity was measured daily and maintained to 3 ppt to prevent further proliferation of parasites and fungal infections for the duration of the experiment. Fish were fed ad libitum with 10-mm sinking pellets daily, and also provided with chironomid larvae (bloodworms, thawed from frozen stock) in their holding tanks haphazardly during the week to maintain familiarity with this food item between trials.

#### Functional response experiments

During the first series of experiments, conducted in 2021, 16 Tench (n = 8 for each temperature treatment) and 12 White Suckers (n = 7, 1 dead individual removed for 18°C treatment and n = 5, 3 dead individuals for 25 °C treatment) were tested. In these trials, individual fish were presented with one of 9 densities of prey (chironomid larvae; 2, 4, 8, 16, 32, 64, 128, 384, 500) every week following a randomized schedule, so that individuals were subjected to every prey density while preventing an effect of acclimation time on consumption of prey. In a second series of experiments conducted in 2023, 15 Tench (n = 7, 1 dead individual at 18 °C; n = 8 at 25 °C) and 12 Brown Bullheads (n = 8 at 18 °C; n = 4, 4 dead individuals at 25 °C) were tested. In the experimental trials, individual fish were presented with one of 7 densities of prey (4, 8, 16, 32, 64, 128, 384) every week following a randomized schedule just as described for the 2021 experiment.

In both series of experiments, individual fish were placed in 150-L experimental tanks (0.6 × 0.6 × 0.4 m) 24 h prior to the experiment and starved to standardize hunger levels. Prey items were frozen chironomid larvae (Hikari® Jumbo Blood Worms (mean ± SD: 19.7 ± 2.04 mm, 31.2 ± 2.44 mg), thawed before use. All three species of fish include chironomid larvae as common prey in their diets (Klarberg and Benson 1975; Keast 1985; Petridis 1990; Giles et al. 1990; Hayes et al. 1992; Kline and Wood 1996; Saint-Jacques et al. 2000). Fishes were then subjected to the determined prey density for 3 h after which they were returned to their holding tanks and uneaten prey were counted. Controls were run where prey would be introduced in experimental tanks with no fish, and then counted 3 h later in order to account for any loss of prey via the drainage systems; as retrieval rate was near 100% (99.7% in 2021, 99.4% in 2023), any loss via drainage was deemed negligible. Individuals were given 7 days of rest between trials. The first set of experiments were conducted between October 5, 2021, and December 21, 2021. Temperature in experimental tanks was controlled using the same system as in the holding tanks (17.79 ± 0.01 °C; 24.97 ± 0.02 °C). The second set of experiments were conducted between January 30 2023, and April 4, 2023, and temperatures were 18.31 ± 0.01 °C for the 18 treatment and 25.37 ± 0.05 °C for the 25 °C treatment. After the end of experiments, fish were euthanized, measured and weighed, and their sex was determined.

#### Body condition factors

At the start and end of the experiments, we weighed individual fish to the nearest 0.1 g, measured their total length (mm), assessed their sex, and checked for lesions and parasites. At the end of the experiment we removed and weighed their livers to the nearest 0.001 g, and weighed them again after drying them in a drying chamber (Binder® model ED23) at 60 °C until constant mass (minimum 48 h).

### Data analysis

#### Functional response curves and metrics

Prior to analyses, we excluded two types of data: (1) individuals that never consumed prey items across any experimental trials (complete non-feeders), and (2) specific trials where an individual failed to consume prey in the first iteration, which we then repeated—in these cases, we excluded the failed first iteration and retained the successful repeated trial. Initial body mass homogeneity among treatment groups was confirmed using one-way ANOVA (*p* > 0.05).

Functional response curves were evaluated using the *frair_test*, a function from the *frair* package that evaluates the sign of the first-order term of a polynomial logistic regression on the proportion of prey (Juliano 2001), such that a type II curve is indicated if the term is negative, and a type III is indicated if the term is positive.

Functional response data were analyzed in R version 4.1.2, following Pritchard et al. (2017). As prey were not replaced during experiments, type II curve fitting is based on the Rogers-Royama random predator equation (Royama 1971; Rogers 1972):

1 $$ N_{e} = N_{0} \cdot \left( {1 - e^{{a \cdot \left( {N_{e} \cdot h - T} \right)}} } \right) $$

where N_e_ is the number of prey eaten, N_0_ the initial number of prey supplied, *a* the attack rate, *h* the handling time and *T* the experimental time. A type III curve is fitted based on Hassell’s (1977) equation, which follows Rogers’ formula but where $a=\frac{b\bullet {N}_{0}}{1+c\bullet {N}_{0}}$, with *b* and *c* being coefficients to be fitted.

Subsequently, we used *frair_fit* function to estimate attack rate *a* (in Type II curves only) and handling time *h* with the fit of the preferred model. Attack rates were compared using *frair_compare* function between Type II responses only, since it is density-dependent in type III curves, and changes along the gradient of prey density. Maximum feeding rate (MFR; *T/h*) was used to compare the asymptote of functional response curve for each treatment. We plotted the bootstrapped values of fitted parameters (N = 999) for each treatment and compare the 95% CI of consumption values across the gradient of prey densities. Lack of overlap between the bootstrapped confidence intervals indicates significant difference.

To compare the impact potential of the three species, we calculated the Functional Response Ratio (FRR), i.e. *a/h* (Cuthbert et al. 2019) and the Relative Impact Potential (RIP) metric (Dick et al. 2017b), which is the product of the ratio of maximum feeding rates (MFR) and abundances (AB) of an invasive species and a native comparator:

2 $$ RIP = \left( {\frac{{MFR_{invasive} }}{{MFR_{native} }}} \right) \times \left( {\frac{{AB_{invasive} }}{{AB_{native} }}} \right) $$

In our study, standardized abundance datasets for both Tench and the native species were not available. Consequently, we sought to determine the abundance ratio required for the RIP to achieve equilibrium, signifying an equal ecological impact between the invasive and native species. With RIP equating to 1, the second term of the equation can be solved as follows:

3 $$ \left( {\frac{{AB_{invasive} }}{{AB_{native} }}} \right) = \left( {\frac{{MFR_{native} }}{{MFR_{invasiveive} }}} \right)^{{}} $$

where the MFR ratio can be used to estimate the abundance ratio at which both invasive and native population will have comparable impact on prey population.

To evaluate the relative change of functional response parameters, we used a *log percentage* (L% = 100 log_e_(*x*/*y*), Tornqvist et al. 1985). Relative change in magnitude is the same regardless of the direction of comparison, allowing for clearer and more consistent reporting of relative change between functional response curve parameters.

#### Body condition factors

We calculated relative condition factors (Le Cren 1951) $K=\frac{W}{a\bullet {L}^{b}}$, using allometric parameters derived from length–weight relationships of populations in the St. Lawrence River (Supplementary table 1). We then compared condition factors of individuals before and after experiments for each treatment. In the 2023 set of experiments, we added the following metrics of condition; we calculated hepatosomatic index (HSI) using the following formula,$HSI= \frac{WML}{FTM-WML} \times 100$, where WML is the wet mass of liver in grams, and FTM is the fish total mass in gram including the liver (Wuenschel et al. 2019). We compared HSI among temperature treatment of the same species. We calculated percentage of the dry mass of the liver (%DML) following Wuenschel et al. (2019),$\%DML=\frac{DML}{WML}\times 100$, where DML means dry liver mass. For relative condition factors, paired t-tests were conducted to compare values between the start and end of the experimental period within each treatment group, with Holm correction for multiple comparisons. For HSI and DWL, which were measured only at the end of the experiment, independent t-tests were performed to compare temperature treatments within each species (18 °C vs 25 °C), also with Holm correction. Statistical significance was set at α = 0.05.

## Results

### Functional responses

**Fig. 1:**
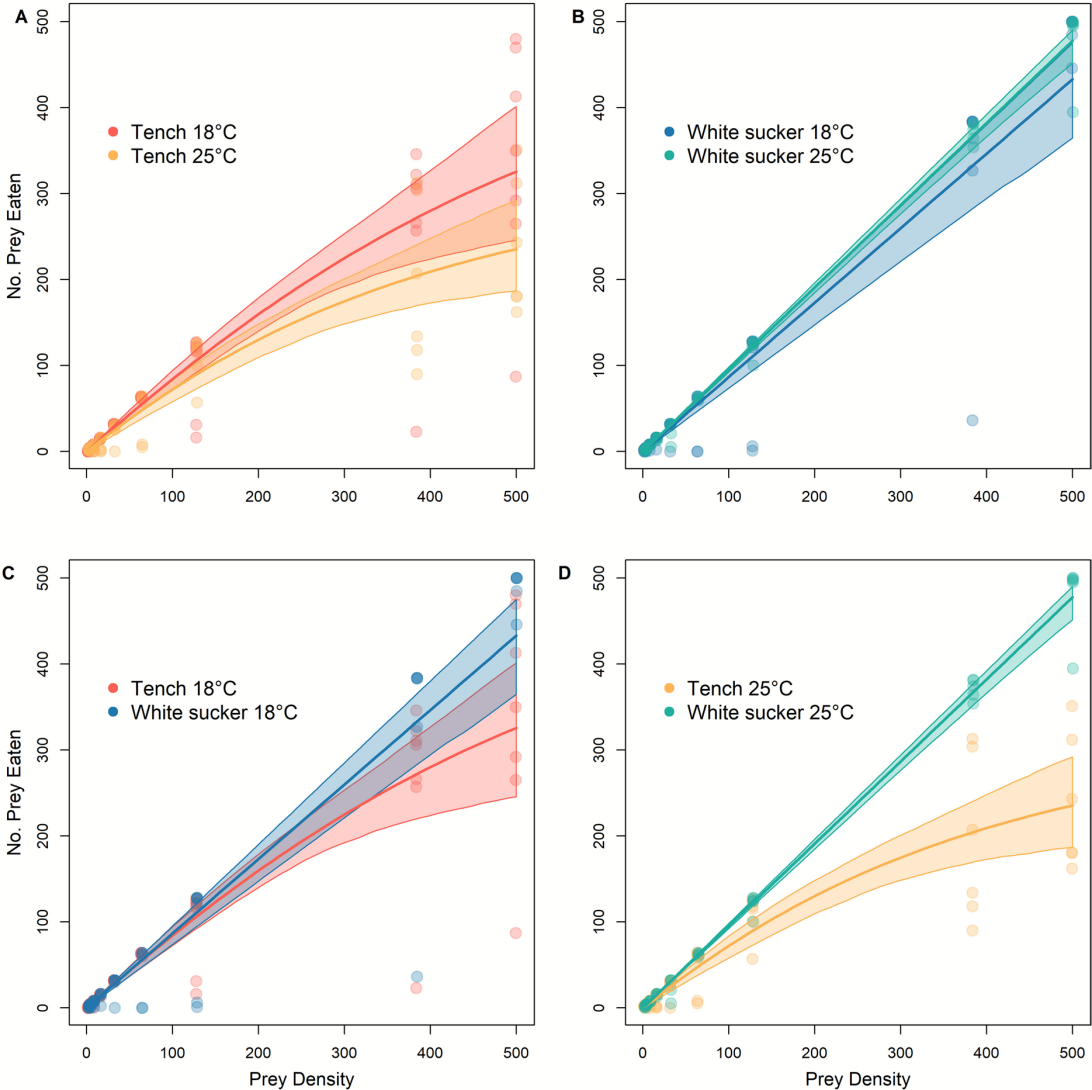
First experiment (2021). Comparative functional response curves of Tench and White Sucker at two temperature treatments (18°C and 25°C). Shaded areas represent 95% confidence interval of curve parameters from bootstrapped values (N = 999). The lines represent the fitted models and the circles are experimental trials

In the first set of experiments, Tench functional response curves were fit using the Type II model, while White Sucker functional response could not be fitted to a curve type due to the absence of inflection and asymptote in their functional response (Table 1). Maximum feeding rates of Tench are higher at 18 °C than at 25 °C. While there is overlap in bootstrapped confidence intervals (Fig. 1A), model estimates for handling time (*h*) were lower at 18 °C than at 25 °C (*∆h* = 2. 90 × 10^–3^ ± 4.20 × 10^− 4^, *p* < 0.001), as reflected in estimated maximum feeding rates of 647 and 398 prey at 18 °C and 25 °C, respectively (Table 2). Attack rate (*a*) for Tench was also significantly higher at 18 °C than 25°C (*∆a* = 0.19 ± 0.03, *p* < 0.001, Table 2). In contrast, maximum feeding rates for White Sucker were too high to be estimated over the range of prey provided, and their consumption rates over this range did not differ between temperature treatments (Fig. 1B). Based on bootstrapped curves, White Sucker consumption rates were higher in both temperature treatments compared to Tench; however, a comparison of curve parameters was not possible with the absence of an inflection in the White Sucker functional response (Fig. 1C, 1D).

**Table 1:** Results *frair_test* for determining best fit (Type II or Type III) for the functional response curve at each treatment for 2021 (top) and 2023 experiments (bottom). The Juliano method classifies curves as Type II or Type III, if the first order term is negative or positive, respectively

Species	Temp (°C)	1st order term, p
Tench	18	− 0.002, 2.2 × 10^− 16*^
	25	− 0.003, 2.2 × 10^− 16*^
White Sucker	18	—
	25	0.008, 8.1 × 10^− 6*^
Tench	18	− 0.004, 2.2 × 10^− 16*^
	25	− 0.006, 2.2 × 10^− 16*^
Brown Bullhead	18	− 0.004, 2.2 × 10^− 16*^
	25	− 0.004, 2.2 × 10^− 16*^

**Table 2:** Parameter estimates for each Functional Response experimental group in 2021 (top part of table) and 2023 (bottom) (± standard error, SE) derived with the *frair_fit* function. Parameter *a* is the attack rate and *h* is the handling time of functional response curve. MFR is maximum feeding rate and FRR is the functional response ratio with spread values range in parentheses. Asterisks indicate statistical significance

Species	Temp (°C)	Fit used	*a* ± SE, p	*h* ± SE, p	MFR (T/h)	FRR (*a/h*)
Tench	18	Rogers II	0.71 ± 0.02, 2.20 × 10^− 16*^	4.6 × 10^− 3^ ± 2.3 × 10^− 4^, 2.20 × 10^− 16*^	647	153 (140–167)
	25	Rogers II	0.52 ± 0.02, 2.20 × 10^− 16*^	7.5 × 10^− 3^ ± 3.4 × 10^− 4^, 2.20 × 10^− 16*^	398	69 (63–75)
White sucker	18	Rogers II	—	—	—	—
	25	Rogers II	—	—	—	—
Tench	18	Rogers II	2.04 ± 0.09, 2.20 × 10^− 16*^	0.004 ± 0.000, 2.20 × 10^− 16*^	285	581 (528–644)
	25	Rogers II	1.33 ± 0.07, 2.20 × 10^− 16*^	0.012 ± 0.001, 2.20 × 10^− 16*^	87	116 (105–128)
Brown bullhead	18	Rogers II	1.27 ± 0.05, 2.20 × 10^− 16*^	0.005 ± 0.000, 2.20 × 10^− 16*^	209	265 (244–288)
	25	Rogers II	0.59 ± 0.05, 2.20 × 10^− 16*^	0.013 ± 0.001, 2.20 × 10^− 16*^	75	44 37–53)

**Fig. 2:**
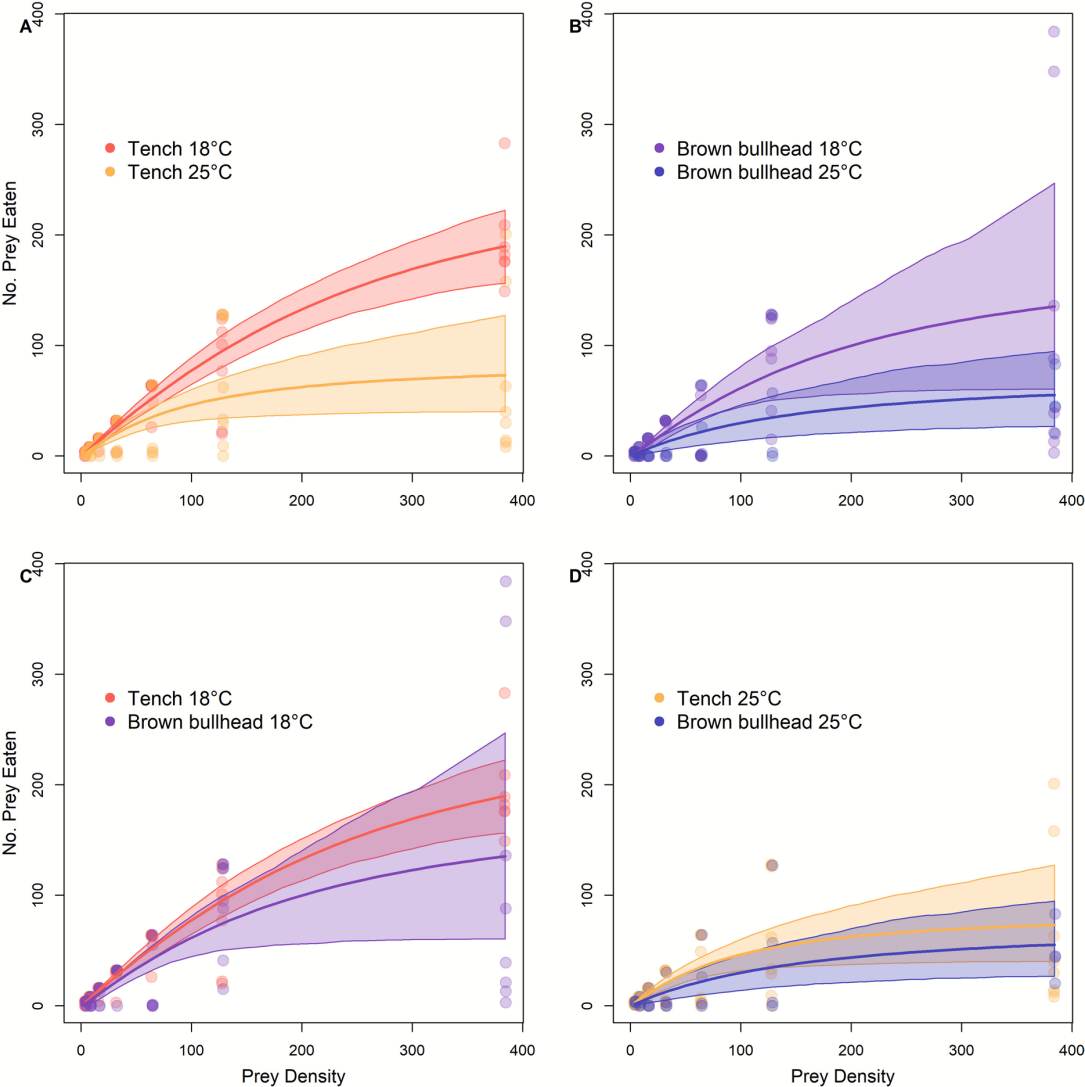
Second experiment (2023). Comparative functional response curves of Tench and Brown Bullhead at two temperature treatments (18°C and 25°C). Shaded areas represent 95% confidence interval of curve parameters from bootstrapped values (N = 999). The lines represent the fitted models and the circles are experimental trials

In the second set of experiments, functional response curves of both species and temperature treatments were fitted using a type II model (Table 3). Tench exhibited a higher feeding efficiency at 18°C than 25°C across most of the range of prey; their attack rate and handling time are significantly higher and lower, respectively, at 18°C (*∆a* = 0.709 ± 0.114, *p* < 0.001; *∆h* = 0.008 ± 0.001, *p* < 0.001; Table 3; Fig. 2A) than at 25°C. Brown Bullhead functional response curves overlap with bootstrapped model, but their attack rate and handling time are also significantly higher and lower, respectively at 18 °C (*∆a* = 0.678 ± 0.074, *p* < 0.001; *∆h* = 0.009 ± 0.001, *p* < 0.001; Table 3, Fig. 2B) than at 25 °C. Model estimates comparisons for *a* (*∆a* = 0.772 ± 0.101, *p* < 0.01) and *h* (*∆h* = 0.001 ± 0.000, *p* < 0.01) (Table 3) parameters were, respectively, significantly higher (*a*) and lower (*h*) for Tench compared to Brown Bullhead at 18 °C, although confidence interval of bootstrapped curves were overlapping (Fig. 2C). At 25 °C, maximum consumption was not significantly different between Tench and Brown Bullhead (*∆h* = 0.001 ± 0.001, *p* = 0.16), but Tench attack rate was significantly higher (*∆a* = 0.742 ± 0.090, *p* =  < 0.01) than Brown Bullhead (Table 3; Fig. 2D). From 18 to 25 °C in the first set of experiments, Tench attack rate (*a*) decreased by ~ 31 log percent (L%) and handling time (*h*) increased by 155 L% (Table 3). When comparing parameters from 18 to 25 °C in the second set of experiments we saw a decrease of 119 L% for the attack rate and an increase of 43 L% for the handling time. For Brown Bullhead, attack rate decreased by 76 L% and handling time increased by 102 L%.

**Table 3:** Parameter comparisons (*a* = attack rate, *h* = handling time) in absolute values between functional response curves using *frair_compare* for 2021 (top part of table) and 2023 (bottom) with associated Standard Error and p value. Dashes indicate absence of comparison due to poor fit of functional response to one of experimental treatments

Treatment comparison	*∆a* ± SE, p	*∆h* ± SE, p	Fit used
Tench 18 °C—Tench 25 °C	0.19 ± 0.03, 2.20 × 10^− 16*^	2. 90 × 10^− 3^ ± 4.20 × 10^− 4^, 2.20 × 10^− 16*^	Rogers II
Tench 18 °C—White Sucker 18 °C	—	—	—
White Sucker 18 °C—White Sucker 25 °C	—	—	—
Tench 25 °C—White Sucker 25 °C	—	—	—
Tench 18 °C—Tench 25 °C	0.709 ± 0.114, 2.2 × 10^− 16*^	0.008 ± 0.001, 2.2 × 10^− 16*^	Rogers II
Tench 18 °C—Brown Bullhead 18 °C	0.772 ± 0.101, 2.2 × 10^− 16*^	0.001 ± 0.000, 2.2 × 10^− 16*^	Rogers II
Brown Bullhead 18 °C—Brown Bullhead 25 °C	0.678 ± 0.074, 2.2 × 10^− 16*^	0.009 ± 0.001, 2.2 × 10^− 16*^	Rogers II
Tench 25 °C—Brown Bullhead 25 °C	0.742 ± 0.090, 2.2 × 10^− 16*^	0.001 ± 0.001, 0.162	Rogers II

The FRR values varied across experimental groups as follows in descending order: Tench at 18 °C (582), Brown Bullhead at 18 °C (265), Tench at 25 °C (116) and finally Brown Bullhead at 25 °C (44) (Table 2).

At 18 °C, the determined abundance ratio for reaching a Relative Impact Potential (RIP) equilibrium (reflecting equivalent ecological impact between invasive and native species) was 0.73, with range of values 0.66–0.80 when spreading the uncertainties in maximum feeding rates. At 25 °C, the ratio of abundance was 0.86 (0.75–0.99).

### Body condition factors

**Fig. 3:**
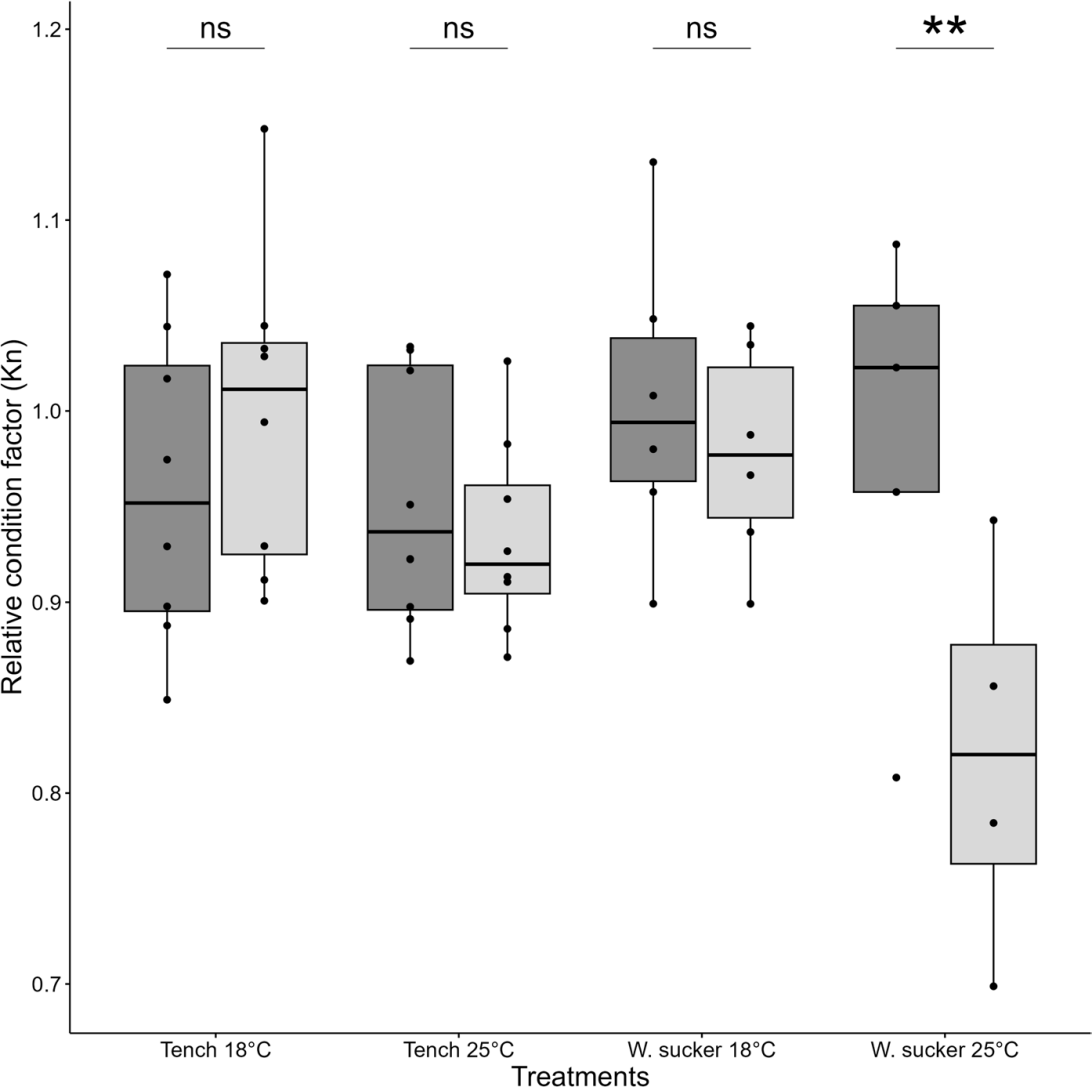
Comparison of relative condition factors (K_n_) of Tench and White Sucker before (dark grey) and after (light grey) being subjected to functional response experiments at two different temperatures (18°C and 25°C). The black points represent condition factors of individual. Black line represent median value, box extremities 25th and 75th quantiles. Boxplot whiskers represent data points range excluding outliers. Asterisks shows significance with the following pattern: < 0.05*; < 0.01**, < 0.001***

In the Tench-vs-White Sucker comparison, experimental groups mean weights were not significantly different from each other (ANOVA, *F*(3, 23) = 0.671, *p* = 0.579; Table 4). White Sucker at 25 °C was the only experimental group showing a significant decline in relative condition factors post-experiment (∆K_n_ = − 0.17, t = 6.04, *p* = 0.009; Fig. 3), consistent with our third prediction for reduced condition when outside the optimal thermal range. All other treatment groups showed no significant difference in condition before and after experiments.

**Table 4:** Mean and standard deviation of weights for the functional responses and body condition factors groups in 2021 (top) and 2023 (bottom) and their associated one-way ANOVA statistics

Experimental group	Start mean weight (g) ± σ	ANOVA start	End mean weight (g) ± σ	ANOVA end
Tench 18	723.3 ± 75.8	*F*(3, 23) = 0.671, *p* = 0.579	761.0 ± 102.0	*F*(3, 22) = 6.589, *p* = 0.002
Tench 25	717.0 ± 94.4		690.3 ± 90.6	
White Sucker 18	686.3 ± 126.0		671.2 ± 101.6	
White Sucker 25	649.9 ± 112.1		496.3 ± 96.8	
Tench 18	318.2 ± 52.6	*F*(3,27) = 0.156, *p* = 0.925	326.9 ± 81.6	*F*(3, 23) = 4.766, *p* = 0.01
Tench 25	307.9 ± 30.4		281.2 ± 37.4	
Brown Bullhead 18	315.4 ± 15.5		378.8 ± 24.1	
Brown Bullhead 25	311.5 ± 16.3		325.3 ± 50.4

**Fig. 4:**
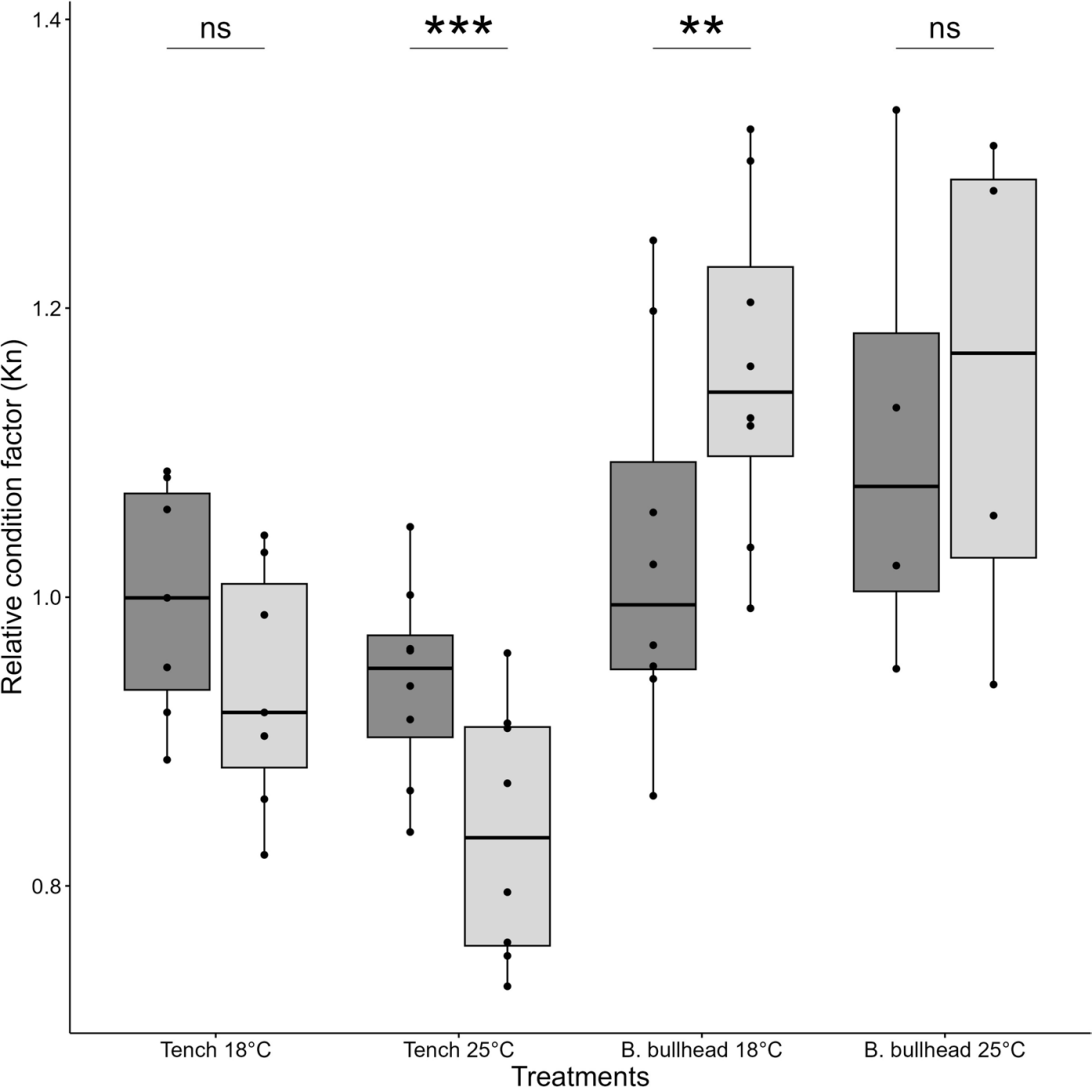
Comparison of relative condition factors of Tench and Brown Bullhead (K_n_) before (dark grey) and after (light grey) being subjected to functional response experiments at two different temperatures (18°C and 25°C). The black points represent condition factors of individual. Black line represent median value, box extremities 25th and 75th quantiles. Boxplot whiskers represent data points range excluding outliers. Asterisk shows significance with the following pattern: < 0.05*; < 0.01**, < 0.001***

**Fig. 5:**
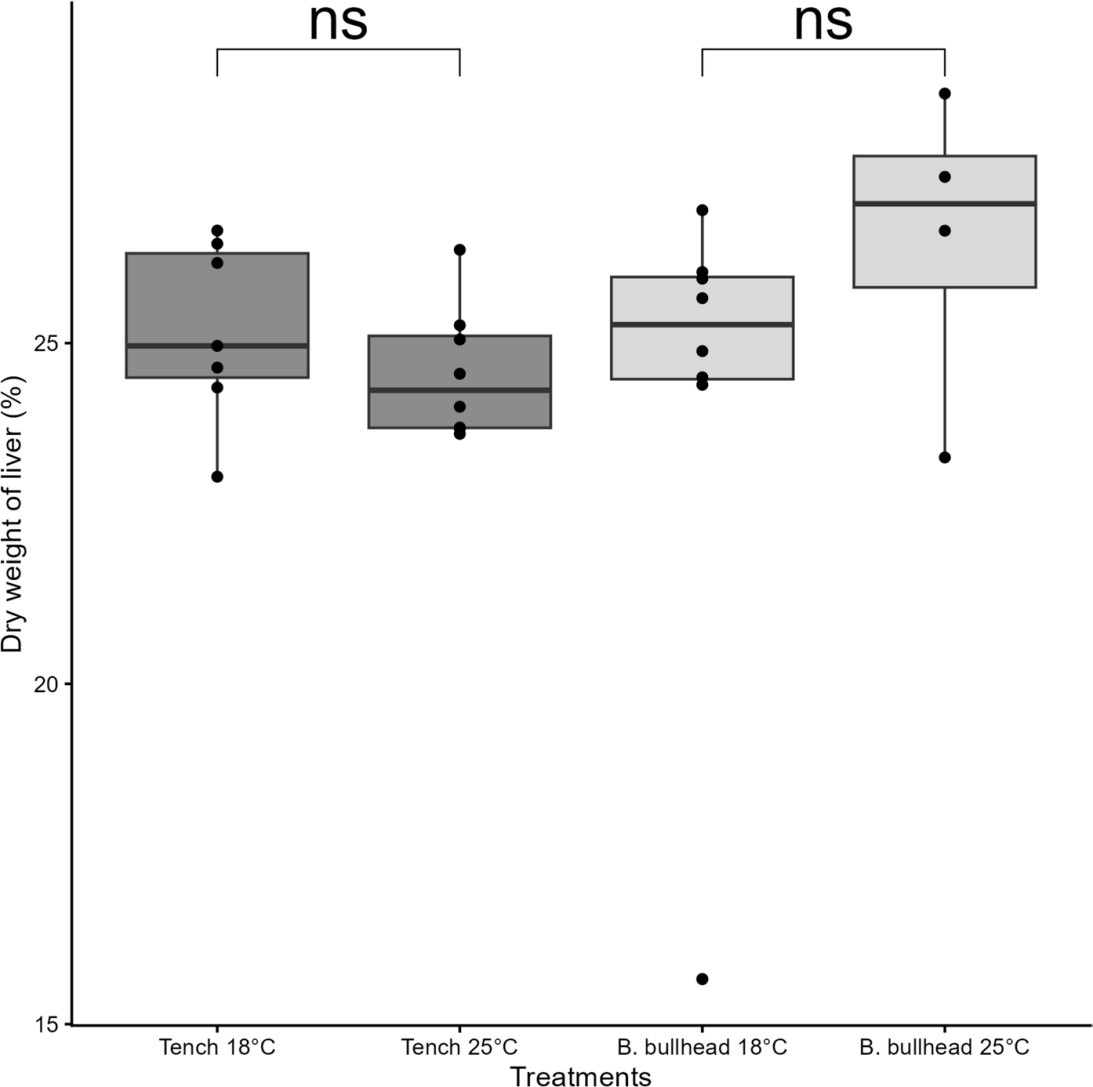
Comparison of percent dry weight of liver (%DWL) comparison of Tench (dark grey) and Brown Bullhead (light grey) after the functional response experiments at 18°C and 25°C. The black points represent condition factors of individual. Black line represent median value, box extremities 25th and 75th quantiles. Boxplot whiskers represent data points range excluding outliers

**Fig. 6:**
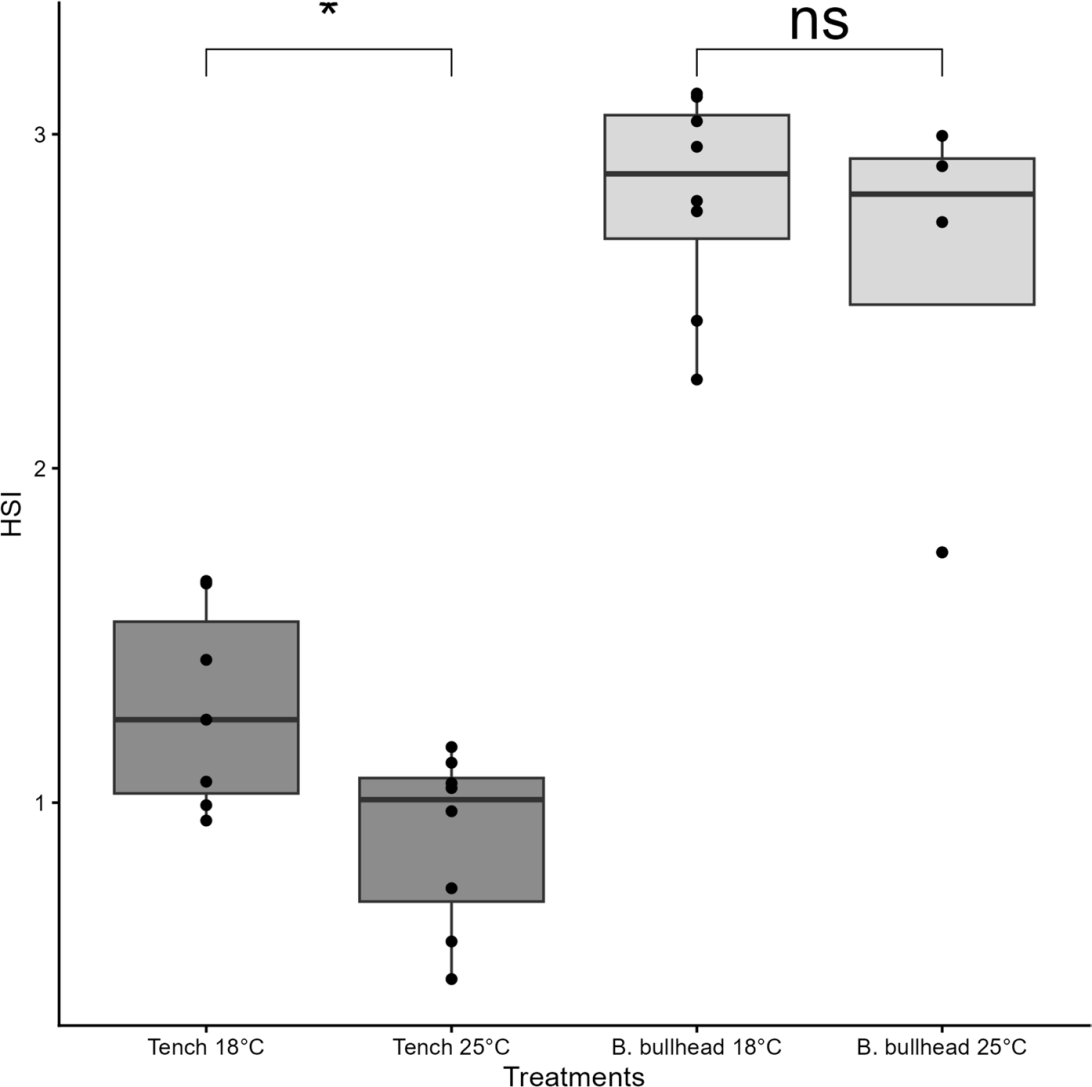
Hepatosomatic Index comparison of Tench (dark grey) and Brown Bullhead (light grey) after the functional response experiments at 18°C and 25°C. The black points represent condition factors of individual. Black line represent median value, box extremities 25th and 75th quantiles. Boxplot whiskers represent data points range excluding outliers. Asterisk shows significance with the following pattern: < 0.05*; < 0.01**, < 0.001***

In the Tench-vs-Brown Bullhead experiment, treatment group mean weights were not significantly different at the start of experiments (ANOVA, *F* = 0.156, *p* = 0.93, Table 4). Tench exposed to temperatures of 25 °C had a significantly lower relative condition factor at the end of the experiment compared to the start (∆K_n_ = − 0.11, *t* = 8.33, *p* < 0.01). Brown Bullhead at 18 °C had a higher relative condition factor condition at the end of the experiment compared to the start (∆K_n_ = -0.13, *t* = − 4.50, *p* < 0.01; Fig. 4). The two other experimental treatments did not result in a difference in condition in the subjected individuals. Comparisons of mean percent dry liver weight (DLW%) revealed no significant difference between treatments of both species (Fig. 5). Intraspecific comparisons of hepatosomatic index (HSI; Fig. 6) at the end of experiments indicate significantly lower HSI values for Tench post-experiment (*t* = 2.65, *p* = 0.02), but no effect for Brown Bullhead between the start and end of experiments (*t* = 0.70, *p* = 0.52).

## Discussion

Feeding efficiency patterns for Tench and Brown Bullhead varied significantly with temperature, whereas White Sucker showed consistently high feeding rates irrespective of temperature. Contrary to our predictions, the anticipated temperature-driven reversal in competitive dominance did not occur at 25 °C; thus, elevated temperature did not give Tench a feeding efficiency advantage over White Sucker under experimental conditions. Specifically, at 18 °C, Tench matched White Sucker consumption over nearly all prey densities, and at 25 °C, Tench maintained comparable consumption up to a moderate prey density (n = 128), suggesting that under current ambient conditions—and in future scenarios with limited resource availability—Tench could still act as a strong competitor against White Sucker.

We recognize that, while these feeding experiments revealed important contrasts, the lack of a completely derived functional response curve for the White Sucker impedes a full comparison of feeding efficiency with Tench and underestimates differences in per capita effects. Superficially, the White Sucker functional response resembles a Type I curve, suggesting that prey handling is negligible (or that fish can handle multiple prey at a time while searching for more prey) and satiation is rarely achieved—conditions long thought to be met exclusively by filter feeding organisms (Jeschke et al. 2004; but see Novak et al. 2025). While we concede the possibility of a White Sucker Type I response, we are aware of no published evidence of such a response found in a benthic fish. A more probable explanation in this case is that the absence of an asymptote is a consequence of an insufficiently high prey density for White Suckers in our experiment. The maximum feeding rate of Tench was significantly higher than Brown Bullhead at 18 °C, indicating superior performance when prey are abundant; but no difference was observed at 25 °C, suggesting that Brown Bullhead exhibits less of a decrease in performance than Tench at preying on chironomid larvae at warmer temperatures. Both Brown Bullhead and Tench exhibited significantly lower consumption at 25 °C compared to 18 °C across nearly the entire range of prey densities tested and at 18 °C, Brown Bullhead showed lower feeding efficiency than Tench, but had an increase in condition factors. This pattern might indicate greater metabolic efficiency in Brown Bullhead at this temperature (Winberg 1960), as they maintained better body condition despite consuming less food. At 25 °C, body condition remained unchanged in both species. Also, mature individuals were used in our first set of experiments to capture the trophic ecology of the longest life stage of Tench, whereas the second set of experiments included smaller individuals, thereby allowing characterization of maximum feeding rates without the burden of excessive enumeration of prey. Therefore, direct comparisons of functional responses between the two experimental sets are not suggested.

Our results differ with those of similar studies testing effect of temperature on functional response. In studies by South et al. (2017) and Mofu et al. (2019), elevated temperatures induced higher maximum feeding rates in predatory fishes, although attack rate was reduced in the fishes studied by Mofu et al. (2019). These discrepancies may reflect methodological differences—most notably, the acclimation period. In our experiments, fish were acclimated to experimental temperatures for 28 days before testing and participated in feeding trials over a seven-week span, simulating more natural, long-term exposure. In contrast, prior studies typically acclimated fish for only 24 h and individuals were not used in multiple trials. Evidence from studies employing extended acclimation times (Avlijaš et al. 2022) similarly found no increase in maximum feeding rates at elevated temperatures, supporting the idea that physiological acclimation and metabolic compensation may explain these contrasting results. A shorter acclimation period may induce acute thermal response (Porter et al. 2022), which can causes an increase in feeding activity related to metabolic activity.

We suspect that the lower feeding rates exhibited by both Tench and Brown Bullhead at higher temperatures are the result of such thermal compensation. Metabolic laws stipulate that biochemical reaction rates increase with acute temperature, thereby raising the energetic demands of ectotherms (Alfonso et al. 2021). However, when subjected to prolonged elevated temperatures, thermal acclimation in fish has been expressed across taxa and habitats as better heat tolerance, increased swimming performance, and a restoration of aerobic scope to levels preceding thermal stress (Sandblom et al. 2016; Nyboer and Chapman 2017; Lapointe et al. 2018; Fu et al. 2018; Christensen et al. 2021). Lower maximum feeding rates observed for Tench and Brown Bullhead after they were acclimated at elevated temperatures suggest a thermal compensation mechanism (aerobic scope protection; Jutfelt et al. 2021), where food ingestion is reduced to limit the increase of postprandial metabolic rate by lowering metabolic-demanding processes. This mechanism maintains the aerobic scope to allow for other aerobic functions. Thermal compensation has been observed in other large eurythermic cyprinids (Ferreira et al. 2014). In our results, this is supported by Tench and Brown Bullhead showing similar responses to the elevated-temperature treatment, and reduced MFR in their optimal temperature range. However, White Sucker did not show this pattern, possibly due to a lesser capacity to metabolically compensate; although the pattern might have been obscured by the absence of a quantifiable maximum feeding rate. Despite their high feeding rates, individual White Suckers in warmwater treatments could not maintain their weight throughout the experiments, perhaps because of elevated metabolism and increased energy expenditures. No loss in body condition was observed in Tench during the first set of experiments, suggesting a higher thermal compensation ability compared to White Sucker. Given that thermal physiology is increasingly recognized as a mediator of both invasion success (Kelley 2014) and impact (Iacarella et al. 2015), we suggest that it should be considered alongside resource consumption efficiency when predicting the impacts of non-native fishes. In our second set of experiments, Tench subjected to elevated temperatures exhibited the reverse pattern: its relative body condition worsened over the span of the experiments and its HSI was lower, although its dry weight of liver was comparable to Tench in low temperature treatment—meaning that liver accounted for less in the whole body weight of Tench, but maintained its energy density. The differential response in body condition of Tench between the two experimental sets might be explained partly by a difference in capture period. In the first sampling period, Tench were caught in temperatures close to experimental conditions and in the summer (19–21 °C; see Methods), but in colder temperatures during the second sampling period and in the fall (14–16 °C). The greater difference in temperatures between capture and holding facilities might have affected Tench thermal acclimation capacity. However, Brown Bullhead did not exhibit signs of negative impact from the difference in temperature between capture and holding facilities, despite being subjected to a greater temperature difference.

For all species in both experiments, temperature affected handling time more than attack rate, with the relative change in handling time being consistently greater. These results align with a meta-analysis showing that temperature affects handling time more than attack rate in fishes (Englund et al. 2011), which suggests that temperature-induced changes in feeding efficiency are driven more by physiological processes affecting digestion and satiation than by behavioral responses affecting search and attack rates. The attack rate parameter encompasses behavioral responses of both predator and prey to temperature, and differential thermal limits between them may create trade-off scenarios (Englund et al. 2011).

Thus, to avoid confounding effects of differential thermal responses between predator and prey, we used thawed dead prey (chironomid larvae, kept frozen prior to experimental trials). However, feeding efficiency can vary with prey type (Haddaway et al. 2012), and in the field, predator–prey interactions are mediated by the thermal acclimation capacity of the interacting species (Grigaltchik et al. 2012). Therefore, a more accurate risk assessment of the trophic impacts of Tench will require experiments that incorporate live prey, in addition to other mediating factors such as prey switching (Cuthbert et al. 2018; McCard et al. 2021) and physical habitat complexity (Santos et al. 2013; Alexander et al. 2015). The ecomorphology of invasive fishes also has added predictive value (Luger et al. 2020). The morphology of the jaw and oral gape influences prey capture success or efficiency, reflected by prey selection and foraging habitat preference (Shadwick 2006). The White Sucker has a sub-terminal mouth adapted to foraging for benthic macroinvertebrates, whereas the Tench and Brown Bullhead mouths are terminal, reducing their efficiency when foraging amongst the sediments but allowing it to be more opportunistic in pelagic habitats or on submerged structures (Helfman et al. 2009). This could partly explain White Sucker absence of inflection in their functional response compared to the two other species.

A differential capacity in thermal adaptation between an invasive fish and functionally similar native species has important ecological implications. Energy storage in fish is linked with higher reproductive success through higher investment in gamete production (Lindström 1998; Marshall et al. 1999; Brosset et al. 2016), higher overwintering survival (Pangle et al. 2004) and lower rates of parasitic infections (Neff and Cargnelli 2004). Furthermore, the reproduction period of each species could conceivably accentuate the effect of a reduced condition factor. Being early spring spawners (starts spawning when temperature reaches 10 °C; Scott and Crossman 1973; Swanson et al. 2021), White Suckers do not have the opportunity to increase energy reserves in the winter months before the spawn (Fernandes and McMeans 2019). The cumulative effect of lower energy accumulation in suboptimal temperatures during warmer months and overwintering may reduce their reproductive success. Tench spawning begins at temperatures near 20 °C (Cudmore and Mandrak 2011) and thus would occur in June or July in the Great Lakes basin, which allows for energy acquisition from substantial prey resources both before and after spawning. Brown Bullhead spawning period mostly overlaps with Tench in late June with reported spawning temperatures ranging from 21 to 25 °C in Canada and northern states of the US (Scott and Crossman 1973; Becker 1983; Jenkins and Burkhead 1993); thus it should not be disadvantaged in terms of energy acquisition prior to spawning. Furthermore, as warming continues, White Suckers might avoid thermally stressed (nearshore, shallow) habitats and migrate to cooler, deeper habitats, where benthic macroinvertebrate biomass can be up to several orders of magnitude lower (Saint-Jacques et al. 2000). However, Brown Bullhead should be able to forage in the same areas as Tench; its environmental tolerances are comparable to Tench and it exhibits similar strategies when subjected to unfavourable conditions (Becker 1983), and thus should not be excluded from high macroinvertebrate-density areas. Our results support the view that as surface water temperatures increase, native fishes with narrower thermal tolerance and plasticity will risk reduced fitness or habitat exclusion, even when competitive abilities are maintained.

For Tench and Brown Bullhead, the functional response ratio (FRR) was higher in the lower temperature treatment than the elevated temperature treatment, and it was higher for Tench than Brown Bullhead for each temperature treatment. Results from our adapted RIP metric revealed nuances of the effects of temperature on Tench and Brown Bullhead. The ratio of abundance values for a RIP value of 1 was higher at 25 °C than 18 °C, indicating that greater numbers of Tench are required to achieve the same magnitude of impact on prey populations as Brown Bullhead at elevated temperatures. It suggests that the competitive edge that Tench holds at lower temperatures declines in warmer temperatures. However, the ratio still has a value of less than 1, indicating a higher *per capita* effect of Tench than Brown Bullhead at both temperatures. This metric does not consider interaction and multiple-predator effects on predation. The feeding efficiency of predators has been shown to be lower than expected when multiple predators are used in a functional response experiment, resulting in a reduction in predation pressure on the prey population. Feeding efficiency of a predator is mediated by the presence—and density—of conspecific and heterospecific competitors and the density of prey available (Wasserman et al. 2016). Therefore, careful monitoring of prey population is necessary to quantify the predatory impact of Tench in relation with predation of co-occurring fishes.

Although White Suckers exhibited a consistently higher per capita feeding rate than Tench at both temperature treatments—indicating a potential competitive advantage through resource exploitation, our results suggest that projected ecological impact depends not only on feeding efficiency but also on the relative abundance and the capacity to maintain fitness under climate warming. While Tench may not competitively dominate trophically similar benthic fishes on a per capita basis, it may exert a greater overall impact under climate warming due to its capacity to compensate energetic demands at high temperatures. Unexpected reductions in Tench and Brown Bullhead feeding performance at elevated temperatures suggest complex interactions involving thermal compensation and metabolic demands, underscoring the importance of considering thermal adaptation capacities in predicting ecological consequences. Increases in Tench populations (possibly further facilitated by climate warming), will negatively impact native populations in resource-limited habitats. Given the unexpected performance declines at high temperatures in both invasive and native benthivores, climate warming may shift not only direct competitive outcomes but also cause unpredictable ecosystem impacts through species-specific physiological responses to chronic thermal stress.

## Supplementary Information

Below is the link to the electronic supplementary material.

Supplementary file 1 (DOCX 14 KB)
