# Supplementary material for: Thermal effects on feeding efficiency and body condition in invasive and native benthivorous freshwater fishes
Source: Biol Invasions. 2026 Feb 12;28(2):51. doi: 10.1007/s10530-026-03767-w (PMC12901266; doi:10.1007/s10530-026-03767-w)
Supplement: Supplementary file 1 — (DOCX 14 KB) [file 10530_2026_3767_MOESM1_ESM.docx]

# Supplementary Material

## Supplementary Tables

1. Tench , White Sucker (2021) and Brown Bullhead (2023) St. Lawrence and Richelieu River population length-weight relationship parameters, derived from a log-log linear regression. Parameter *a* is the intercept of the regression, *b* is the slope, *R^2^* is the fraction of variance explained by the model and *n* is the number of individual measurement included in the model. These parameters were used to calculate condition factors (K) using K=W/a·L^b^.

| Species | *a* | *b* | *R^2^* | *n* |
| --- | --- | --- | --- | --- |
| Tench | 2.51×10^-5^ | 2.90 | 0.987 | 116 |
| White Sucker | 5.05×10^-6^ | 3.12 | 0.935 | 53 |
| Brown Bullhead | 5.73×10^-6^ | 3.14 | 0.880 | 54 |
